# The role of etanercept in juvenile dermatomyositis (JDMS) in children

**DOI:** 10.1186/1546-0096-12-S1-P279

**Published:** 2014-09-17

**Authors:** Katherine L Green, Marinka Twilt, Taunton Southwood

**Affiliations:** 1 Paed Rheumatology, Birmingham, UK; 2 NHS, Birmingham, UK

## Introduction

Juvenile dermatomyositis is a rare autoimmune disease characterised by profound muscle weakness in addition to skin lesions, calcinosis, and underlying vasculopathy. Current treatment plans, including methotrexate and corticosteroids, are ineffective in some patients and may be associated with significant adverse events. The benefits and risks of etanercept in JDMS are not well studied.

## Objectives

The aim of this study is to review the benefit and safety of etanercept in JDMS patients.

## Methods

We performed a single centre retrospective analysis of all consecutive JDMS patients treated with etanercept in a tertiary paediatric referral centre. Data collected included clinical and laboratory data, disease duration, the initial dose of etanercept, and other medication details. The outcome was measured by the Childhood Myositis Assessment Scale (CMAS) scored before commencing Etanercept and at 12 months follow-up.

## Results

Seven JDMS patients (5 female) were treated with etanercept. Median age at diagnosis was 64 months (36-103 months). The most frequent symptoms at diagnosis included proximal muscle weakness in all patients, constitutional features in 6, muscle pain in 5, typical skin features in 4, and arthralgia in 3 patients.

Disease duration until etanercept was 35 months (10-60 months). All children were treated with prednisolone and methotrexate prior to commencing etanercept and continued prednisolone (in reducing doses) and methotrexate concurrently.

Median duration on etanercept was 20 months (range 6-85 months). Five children ceased etanercept; three due to a flare of disease activity, one child due to transfer of care, and one child due to disease remission. Two patients still receive etanercept at time of analysis. Two children who ceased etanercept commenced monthly infliximab therapy with marked disease improvement.The median CMAS score before etanercept was 44 (range 41-47), and at 12 months after commencing etanercept the median CMAS was 46 (range 41-53).

## Conclusion

Etanercept did not demonstrate an appreciable or reliable improvement in the disease control of JDMS. Whilst beneficial for some patients, caution should be taken when initiating etanercept for JDMS. Further multicenter studies are necessary to confirm our findings.

## Disclosure of interest

None declared.

